# Sublingual immunotherapy with recombinant Mal d 1 downregulates the allergen‐specific Th2 response

**DOI:** 10.1111/all.13779

**Published:** 2019-04-10

**Authors:** Claudia Kitzmüller, Beatrice Jahn‐Schmid, Tamar Kinaciyan, Barbara Bohle

**Affiliations:** ^1^ Department of Pathophysiology and Allergy Research Center of Pathophysiology, Infectiology and Immunology Medical University of Vienna Vienna Austria; ^2^ Department of Dermatology Medical University of Vienna Vienna Austria

**Keywords:** apple allergen, birch pollen‐related food allergy, early desensitization, pro‐allergic Th2 cells, sublingual immunotherapy

**Abbreviations**
AIT: allergen‐specific immunotherapy
B2M: beta‐2‐microglobulin
BPRFA: birch pollen‐related food allergy
cpm: counts per minute
C_t_: threshold cycle
PBMC: peripheral blood mononuclear cells
r: recombinant
SI: stimulation index
SLIT: sublingual immunotherapy
TBP: TATA box‐binding protein
Tfh: T follicular helper
Th: T helper
Treg: regulatory T cells
TT: tetanus toxoid

To the Editor,

Birch pollen‐related food allergy (BPRFA) is the most prevalent food allergy in adolescents/adults and affects more than 70% of birch pollen‐allergic patients. Following sensitization to the major birch pollen allergen Bet v 1, allergic symptoms to food occur due to immunological cross‐reactivity to homologous proteins. Mal d 1, the Bet v 1‐homologue in apple, is among the most frequent triggers of BPRFA. Allergen‐specific immunotherapy (AIT) with birch pollen extract is established as effective treatment for birch pollinosis. However, its benefit for the concomitant food allergy is controversial. In search of alternative and more efficient treatment options for BPRFA, we conducted a randomized double‐blind placebo‐controlled sublingual immunotherapy (SLIT) study with standardized doses of recombinant (r) Mal d 1 in patients with birch pollen‐related apple allergy. After 16 weeks, patients who received rMal d 1 showed clinical improvements, tolerating higher amounts of the apple allergen in oral challenges and developing smaller wheal sizes in skin prick tests.^1^ Moreover, their Mal d 1‐specific IgG_4_/IgE ratios increased and their post‐SLIT sera contained antibodies with IgE‐blocking activity (manuscript in preparation). None of these changes were observed in the patients who received placebo.^1^ Here, we characterized for the first time the specific T‐cell response to a sublingually administered recombinant food allergen.

We have previously reported a significant downregulation of allergen‐specific T‐cell proliferation already after 4 weeks of SLIT.^2^ Accordingly, we stimulated PBMC from 20 rMal d 1‐treated individuals collected before, at 4 and 16 weeks of treatment with rMal d 1. We also included Bet v 1, as the majority of Mal d 1‐reactive T cells are originally Bet v 1‐specific cells that cross‐react with the apple allergen. Tetanus toxoid (TT) served as control antigen. Figure 1A shows that rMal d 1‐ and rBet v 1‐induced proliferation was significantly decreased at 4 weeks and 16 weeks, whereas TT‐induced proliferation was not affected. No altered proliferative responses to either allergen or TT were observed with PBMC of 19 individuals receiving placebo (Figure 1A). These results confirmed the early decrease in allergen‐specific T‐cell proliferation in SLIT.^2^ Moreover, the results confirmed our expectations deduced from a previous study demonstrating that two sublingual administrations of 50 μg of rMal d 1 reduced proliferative responses to both Mal d 1 and Bet v 1.^3^ Thus, SLIT performed with recombinant apple allergen also suppressed Bet v 1‐specific, cross‐reactive T cells.

**Figure 1:**
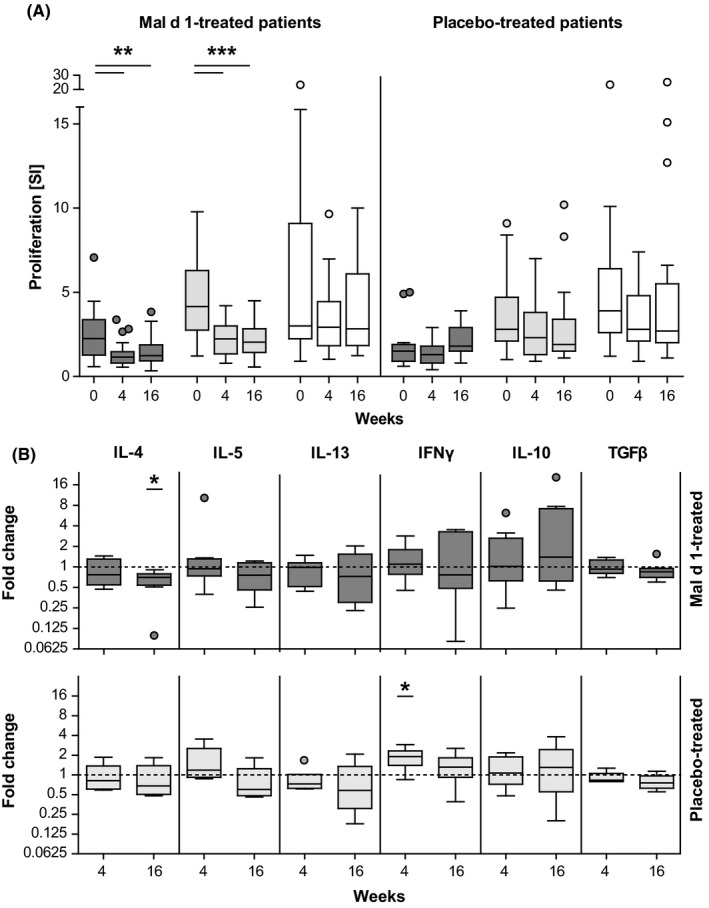
T‐cell responses to specific stimulation. A, PBMC collected from rMal d 1‐ (n = 20) and placebo‐treated (n = 19) individuals before (0), at 4 and 16 wk of treatment were stimulated with Mal d 1 (dark grey), rBet v 1 (light grey) or TT (white). ^3^[H]thymidine uptake was measured as counts per minute (cpm). Stimulation indices (SI) were calculated as the ratio of cpm in cultures plus antigen and cpm in cultures kept in medium alone. B, Relative mRNA expression of cytokines in rMal d 1‐stimulated CD3^+^ T cells isolated from rMal d 1‐treated individuals (n = 8) and individuals receiving placebo (n = 6) at 4 and 16 wk compared with values before treatment. Threshold cycle (C_t_) values were determined; ΔΔC_t_ values (housekeeping genes B2M and TBP) in relation to baseline calculated. Results are depicted as Tukey box plots, and outliers are shown as dots. (*P <* *0.05, **P <* *0.01, ***P <* *0.001, repeated measures one‐way ANOVA with Tukey post hoc test)

To assess the possible induction of Tregs and of a shift from Th2 towards Th1 responses,^2^ we monitored the key cytokines of allergen‐specific Th2, Th1 and Treg cells before and after 4 and 16 weeks of SLIT with rMal d 1. PBMC were stimulated for 6 hours with rMal d 1, CD3^+^ T cells were isolated and mRNA expression levels of IL‐4, IL‐5, IL‐13, IFN‐γ, IL‐10 and TGF‐β were measured by qPCR (see Data S1 for detailed Methods). The post‐treatment to baseline comparison revealed a continuous decline of IL‐4 which was significant at 16 weeks (Figure 1B). IFN‐γ was also slightly decreased at 16 weeks, whereas the other cytokines remained unchanged. These trends of reduced expression of Th2 and Th1 cytokines in the early phase of SLIT matched our findings during SLIT with birch pollen extract 2 and were not observed in the placebo group. The latter showed unchanged levels of all cytokines except for an increase in IFN‐γ after 4 weeks (Figure 1B). To study the allergen‐specific T‐cell response during SLIT more specifically, we sought to characterize the cytokine expression of antigen‐reactive T cells following recently published protocols.^4^ We collected regulatory CD137^+^ and conventional CD154^+^ T cells from rMal d 1‐stimulated PBMC of four rMal d 1‐treated patients by magnetic bead separation and employed QuantiGene Plex expression assay (Thermo Fisher Scientific, Waltham, MA, USA), which uses signal amplification rather than target amplification to detect mRNA levels of up to 80 targets in a single well. However, no signals for the cytokine targets were detected which we refer to limited assay sensitivity due to the extreme rarity of allergen‐specific cells.^4^

It has been suggested that successful AIT induces the selective deletion of so‐called pro‐allergic Th2 effector cells, probably because they are prone to activation‐induced cell death.^5^ These CD27^−^CRTh2^+^CCR4^+^CD4^+^ T cells represent the dominant allergen‐specific T‐cell subset associated with Th2 cytokine production in allergic patients.^5^ In PBMC from rMal d 1‐treated patients, we found a significant decrease in CD27^−^CRTh2^+^CCR4^+^CD3^+^CD4^+^CD45RA^−^ T cells after 4 weeks of treatment, which was even more pronounced after 16 weeks (Figure 2). No changes of pro‐allergic Th2 cells were detected in the placebo group. In parallel, we analysed other T‐cell subsets within CD3^+^CD4^+^CD45RA^−^ memory T cells, that is CCR4^+^ (Th2), CXCR3^+^ and CCR5^+^ (Th1) and CD25^+^CD127^−^ (Treg).^6^ Additionally, we assessed the number of circulating T follicular helper (Tfh)‐like cells (CXCR5^+^), which have been shown to induce Ig production in naive and memory B cells,^7^ and further characterized them as Tfh1 (CXCR3^+^CCR6^−^), Tfh2 (CXCR3^−^CCR6^−^) and Tfh17 (CXCR3^−^CCR6^+^). However, no significant alterations in the relative numbers of any of these subpopulations were observed (Figure S1). We speculate that the proportion of allergen‐specific Th2 cells was sufficient to be detected within the CD27^−^CRTh2^+^CCR4^+^CD4^+^ subset and declined promptly after the onset of SLIT. However, the number of allergen‐specific T cells within the other subsets was too small to result in detectable changes. Still, we cannot exclude that SLIT may have altered the function of Treg cells, for example by upregulation of inhibitory molecules.

**Figure 2:**
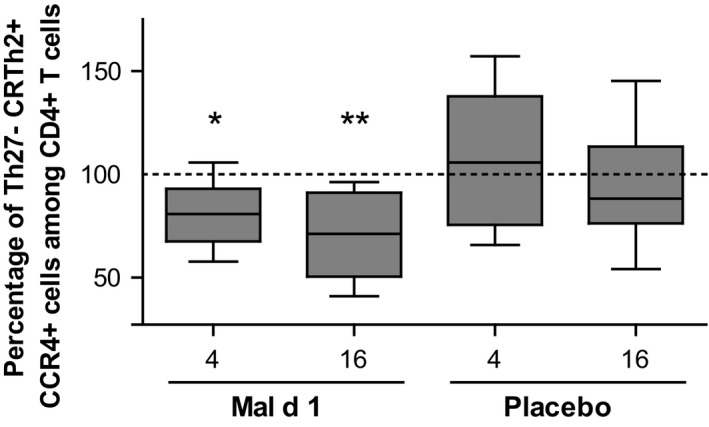
Sublingual immunotherapy reduces pro‐allergic Th2 cells. Percentages of CD27^−^CRTh2^+^CCR4^+^ among CD3^+^CD4^+^CD45RA^−^ T cells in PBMC collected before and after 4 and 16 wk of treatment with rMal d 1 (n = 9) and placebo (n = 8) were determined by flow cytometry. The percentage before treatment was individually normalized to 100. (*P <* *0.05, **P <* *0.01, repeated measures one‐way ANOVA with Tukey post hoc test)

Our longitudinal cellular analyses during 16 weeks of SLIT with standardized daily doses of recombinant apple allergen revealed an early downregulation of the allergen‐specific Th2 response indicated by a significant reduction in allergen‐induced proliferation, IL‐4 synthesis and pro‐allergic T cells. Although deduced from a limited number of available samples and feasible analyses, these findings acquired during sublingual administration of a single allergen accord with previous studies performed with allergen extracts and strengthen the concept that the reconstitution of peripheral tolerance by suppression of allergen‐specific Th2 cells represents an early step in successful SLIT.^2^, ^8^, ^9^ Finally, this study again provides evidence that a switch from Th2 to Th1 responses happens during a later phase of AIT.^6^, ^10^

## CONFLICTS OF INTEREST

The authors declare that they have no conflicts of interest.
