# Supplementary material for: Sublingual immunotherapy with recombinant Mal d 1 downregulates the allergen‐specific Th2 response
Source: Allergy. 2019 Apr 10;74(8):1579–81. doi: 10.1111/all.13779 (PMC6767596; doi:10.1111/all.13779)
Supplement: Supplementary file 1 [file ALL-74-1579-s001.docx]

**ONLINE REPOSITORY**

**Sublingual immunotherapy with recombinant Mal d 1 downregulates the allergen-specific Th2 response**

Claudia Kitzmüller, PhD^1^, Beatrice Jahn-Schmid^1^, Tamar Kinaciyan, MD^2^, and Barbara Bohle, PhD^1^

^1^Department of Pathophysiology and Allergy Research, Center of Pathophysiology,

Infectiology and Immunology, Medical University of Vienna, Vienna, Austria

^2^Department of Dermatology, Medical University of Vienna, Vienna, Austria

**Corresponding author:**

Barbara Bohle

Department of Pathophysiology and Allergy Research

Medical University of Vienna

Waehringer Guertel 18-20

A-1090 Vienna

Phone: +43 1 40400 51140

Fax: +43 1 40400 61880

Email: barbara.bohle@meduniwien.ac.at

**Abbreviations**

B2M, beta-2-microglobulin; cpm, counts per minute; C_t_, threshold cycle; PBMC, peripheral blood mononuclear cells; r, recombinant; SI, stimulation index; TBP, TATA-box binding protein; Tfh, T follicular helper; Th, T helper; Treg, regulatory T cells; TT, tetanus toxoid

**Methods**

**Study design**

Twenty patients were treated with daily administrations of 25 µg of recombinant (r) Mal d 1 and 19 patients with placebo for 16 weeks as described in detail elsewhere (1). Blood was drawn into heparin tubes and PBMC were isolated by centrifugation over Ficoll-Paque (Pharmacia Diagnostics, Uppsala, Sweden). Samples from different time points from the same patient were always analyzed within the same experiment.

**Proliferation assays**

Freshly isolated PBMC (2x10^5^) were incubated in triplicates in 96-well plates (Thermo Fisher Scientific, Waltham, MA USA) with 6.25 or 12.5 µg/ml of rMal d 1 or rBet v 1 (Biomay, Vienna, Austria) in 200 µl of AIM-V medium (Thermo Fisher). Tetanus toxoid (0.25 µg/ml, Calbiochem, Darmstadt, Germany) served as control antigen. After 6 days, proliferation was measured by up-take of tritiated thymidine during the last 16 hours of culture. Stimulation indices (SI) were calculated as the ratio between counts per minute (cpm) obtained in cultures stimulated with allergen and cpm obtained from cultures incubated in medium alone. For each allergen, the concentration inducing optimum proliferative responses before the onset of SLIT was individually determined and used for all subsequent time points.

**Flow cytometry**

Thawed PBMC were rested for 16 hours at 37°C in RPMI 1640 (Sigma Aldrich, Darmstadt, Germany) supplemented with 10% autologous serum before surface marker staining (2-7). Analyses were performed with a FACS Canto II using FACS Diva (BD Biosciences, San Jose, CA, USA) and FlowJo software (TreeStar, Inc., Ashland, OR, USA). The following anti-human antibodies were used: CD3-BV510, CD45RA-FITC (BD Biosciences), CD4-PE/Cy7, CD56-FITC, CXCR5-PE, CD27-PE/Cy7, CD45RA-APC/eFluor780, CRTh2-APC (eBioscience, Thermo Fisher), CXCR3-PerCP/Cy5.5, CCR6-BV421, CD4-BV421, CD25-APC, CD127-BV421, CCR5-Alexa488, CCR4-PE (Biolegend, San Diego, CA, USA).

**Quantitative PCR**

Thawed PBMC (10x10^6^) were incubated in 6 well plates (Corning Costar, Corning, NY, USA) with 5 µg/ml Mal d 1 in 4 ml of RMPI 1640 supplemented with 0.5% human serum albumin (Sigma Aldrich) for 6 hours. Thereafter, T cells were isolated with anti-CD3 Dynal magnetic beads (Thermo Fisher) and subsequently lysed and stored in buffer RLT (QIAGEN, Hamburg, Germany), before RNA was isolated using the QIAGEN RNeasy kit. RNA was reversed transcribed to cDNA using the High-Capacity cDNA Reverse Transcription Kit with RNase Inhibitor (Thermo Fisher). Quantitative real-time PCR was performed with an ABI 7900 HT Sequence Detection System (Thermo Fisher) and cDNA-specific Assays-on-Demand (Thermo Fisher) for IL-4, IL-5, IL-13, IL-10, IFN-γ, and TGF-β in Luna Universal Probe qPCR Master Mix (New England Biolabs, Ipswich, MA, USA). Beta-2-microglobulin (B2M) and TATA-box binding protein (TBP) served as control housekeeping genes. All amplifications were performed in duplicate. The cycle number at which the detected fluorescence exceeded the threshold (C_t_ value) was determined. The fold change induction was calculated by using the softwares SDS 2.4 and DataAssist 2.0 (both Thermo Fisher), comparing the samples collected at 4 and 16 weeks of treatment with before treatment.

**Isolation of allergen-specific conventional and regulatory T cells**

Thawed PBMC (20x10^6^) were rested for 16 hours at 37°C in RPMI 1640 supplemented with 10% autologous serum. Rested cells were re-suspended in 1 ml medium containing 10 µl anti-human CD40 antibody (Miltenyi Biotec, Bergisch Gladbach, Germany) and 0.5 µl anti-human CD28 antibody (Sanquin, Amsterdam, The Netherlands) and stimulated with 20 µg rMal d 1 in 12-well plates (Corning Costar) for 6 hours. Cells were harvested and depleted of monocytes and cytotoxic T cells using anti-CD14 and anti-CD8 Dynal magnetic beads (Thermo Fisher). The remaining cells were labelled using anti-human CD154-biotin and CD137-PE and isolated with MACS Biotin beads and PE beads (all Miltenyi Biotec), respectively, according to manufacturer’s instructions.

**QuantiGene Plex array**

Isolated allergen-specific T cells were lysed using the QuantiGene sample processing kit (Thermo Fisher) according to manufacturer’s instructions. Lysates were used undiluted for the QuantiGene Plex assay following instructions using a customized Plex array containing probes for the targets IL-2, IL-3, IL-4, IL-5, IL-9, IL-10, IL-13, IL-21, IL-22, IFN-γ,TNF-α,TBX21, GATA3, FOXP3 and RORC, as well as the housekeeping genes GAPDH and RPL32. The samples were analyzed on a BioPlex 200 system using Bio-Plex Manager Software (Bio-Rad Laboratories, Hercules, CA, USA).

**Statistical analysis**

Repeated measures one-way ANOVA with Tukey post hoc tests were used to test for statistical significance using IBM SPSS Statistics 24.0 (SPSS, Chicago, IL, USA). Differences were considered significant if P<0.05.

**Figure E1: CD4^+^ memory T cell subsets during the course of SLIT with rMal d 1 or placebo**

**
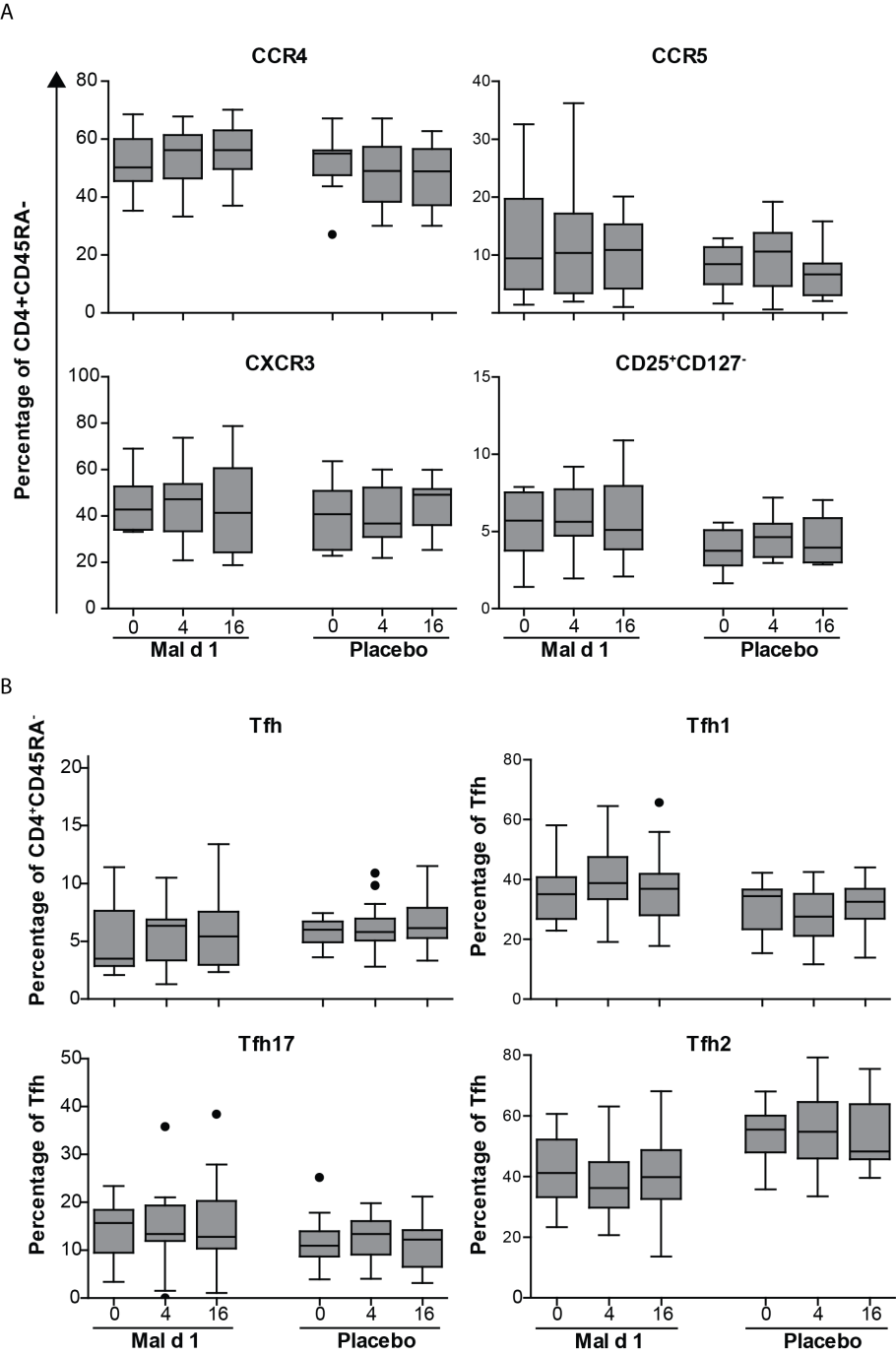
**

CD3^+^CD4^+^CD45RA^-^ memory T cells in PBMC collected before (0) and at 4 and 16 weeks of SLIT with rMal d 1 or placebo (n=9 for both groups) were stained with surface markers specific for A) T cell polarization, i.e. CCR4^+^ (Th2), CCR5^+^ and CXCR3^+^ (Th1) and CD25^+^CD127^-^ (Treg) and B) T follicular helper (Tfh) cells, i.e. CXCR5^+^ and within this population for Tfh1 (CXCR3^+^ CCR6^-^), Tfh17 (CXCR3^-^ CCR6^+^) and Tfh2 (CXCR3^-^ CCR6^-^) subsets. Stainings for T cell polarization were done according to (2-4), for Tfh cells according to (5-7). Percentages of positive cells are shown as Tukey box plots, outliers are shown as dots.

**References**

1. Kinaciyan T, Nagl B, Faustmann S, Frommlet F, Kopp S, Wolkersdorfer M, et al. Efficacy and safety of 4 months of sublingual immunotherapy with recombinant Mal d 1 and Bet v 1 in patients with birch pollen-related apple allergy. *J Allergy Clin Immunol* 2018;**141**(3):1002-1008.

2. Bonvalet M, Moussu H, Wambre E, Ricarte C, Horiot S, Rimaniol AC, et al. Allergen-specific CD4+ T cell responses in peripheral blood do not predict the early onset of clinical efficacy during grass pollen sublingual immunotherapy. *Clin Exp Allergy* 2012;**42**(12):1745-1755.

3. Yamamoto J, Adachi Y, Onoue Y, Adachi YS, Okabe Y, Itazawa T, et al. Differential expression of the chemokine receptors by the Th1- and Th2-type effector populations within circulating CD4+ T cells. *J Leukoc Biol* 2000;**68**(4):568-574.

4. Mahnke YD, Brodie TM, Sallusto F, Roederer M, Lugli E. The who's who of T-cell differentiation: human memory T-cell subsets. *Eur J Immunol* 2013;**43**(11):2797-2809.

5. Morita R, Schmitt N, Bentebibel SE, Ranganathan R, Bourdery L, Zurawski G, et al. Human blood CXCR5(+)CD4(+) T cells are counterparts of T follicular cells and contain specific subsets that differentially support antibody secretion. *Immunity* 2011;**34**(1):108-121.

6. Schmitt N, Bentebibel SE, Ueno H. Phenotype and functions of memory Tfh cells in human blood. *Trends Immunol* 2014;**35**(9):436-442.

7. Wei Y, Feng J, Hou Z, Wang XM, Yu D. Flow cytometric analysis of circulating follicular helper T (Tfh) and follicular regulatory T (Tfr) populations in human blood. *Methods Mol Biol* 2015;**1291**:199-207.
